# Characterizing Dietary Intakes in Rural Australian Adults: A Systematic Literature Review

**DOI:** 10.3390/nu12113515

**Published:** 2020-11-15

**Authors:** Laura Alston, Troy Walker, Katherine Kent

**Affiliations:** 1 The Global Obesity Centre, Institute for Health Transformation, Deakin University, Geelong, VIC 3220, Australia; troy.walker@deakin.edu.au; 2 Faculty of Health, Deakin Rural Health, Deakin University, Warrnambool, VIC 3280, Australia; 3 Colac Area Health, Colac, VIC 3250, Australia; 4 Centre for Rural Health, College of Health and Medicine, University of Tasmania, Launceston, TAS 7250, Australia; Katherine.kent@utas.edu.au

**Keywords:** rural, nutrition, community, dietary assessment

## Abstract

Rural Australians experience a higher burden of diet-related chronic disease than their metropolitan counterparts. Dietary intake data is needed to understand priorities for nutrition initiatives that reduce disparities in the health of rural Australians. A systematic literature review aimed to synthesize the evidence on dietary intakes in adult populations residing in rural and remote Australia, to identify areas for intervention, and make recommendations for future research. A comprehensive search of five electronic databases was conducted and 22 articles were identified for inclusion. Half of the included studies (50%) collected dietary data using non-validated questionnaires and nearly half (41%) did not benchmark dietary intakes against public health guidelines. Most studies (95%) showed that rural populations have suboptimal dietary intakes. Despite the high level of preventable diet-related disease in rural and remote Australia, this review identified that there is insufficient high-quality dietary data available and a lack of consistency between dietary outcomes collected in research to inform priority areas for intervention. Further cross-sectional or longitudinal data should be collected across all remoteness areas, using robust, validated dietary assessment tools to adequately inform nutrition priorities and policies that reduce rural health disparities.

## 1. Introduction

Dietary risk factors, namely, low intakes of fruits, vegetables, and whole grains and high intakes of sodium and saturated fat, are now the leading preventable risk factors contributing to the burden of disease in Australia [1] and globally [2]. The Australian Burden of Disease study in 2015 showed that 38% of the burden of disease was attributable to preventable risk factors [3] and that dietary risk factors and obesity contributed to almost half of the preventable burden of disease [3]. 

People who live in rural and remote areas experience higher rates of diet-related disease when compared to their metropolitan counterparts, including cardiovascular disease, type 2 diabetes, high blood pressure, chronic kidney disease, and overweight and obesity [1,4]. Access to and promotion of healthy food is challenging in rural and remote Australia as small population sizes and increasing distances from urban centers limits the variety of food available and increases the price of fresh, healthy food [1,5,6]. In addition, Australians living in rural and remote areas experience greater sociodemographic disadvantage than those in urban areas, which makes healthy food more unaffordable at a household level. As a result, national health survey data has shown that only 1 in 10 people living outside major cities reported meeting recommendations for vegetable intake and fewer Australians living in rural areas meet fruit recommendations (47%) than their metropolitan counterparts (52%) [1]. However, the national health survey estimates food and nutrient information for the population based on a 24 h dietary recall, which may be less reflective of habitual dietary patterns than other dietary assessment techniques (such as a food frequency questionnaire (FFQ)). Additionally, the survey includes only limited sampling from rural populations and no sampling in very remote areas of Australia, identifying a clear opportunity to collect additional robust dietary data in these regions to strengthen our understanding of how dietary risk factors are driving rural health inequalities [7]. 

Growing evidence suggests that the gap in mortality from cardiovascular disease between rural and metropolitan Australians would be reduced if improvements in diet could be achieved in rural areas [4,8]. Alston et al. reported that the gap in ischemic heart disease mortality between rural and metropolitan Australia could be reduced by 38% if rural Australians were able to achieve the same risk factor levels for diet, alcohol, physical activity, and tobacco smoking as their metropolitan counterparts, with further gains if all public health recommendations were met [4,8]. This study identified the lack of dietary data collected from rural areas as a major barrier preventing further modelling in all rural and remote areas of Australia. Additionally, a recent review by Alston & Partridge identified a lack of evidence from intervention studies that aimed to improve dietary intake in rural Australia over the past 20 years and a major limiting factor was that dietitians and/or nutritionists were rarely involved in study design or delivery [9]. Given there is a lack of high level investment and progress made in addressing issues with healthcare access in rural and remote areas of Australia, interventions to reduce dietary risk factors are important to support the health of current and future generations. Clear identification of key priority areas for initiatives or policy change to reduce dietary risk factors in rural areas is required, in addition to an understanding of the current dietary intake patterns in these communities. With an absence of routine national-level monitoring of dietary intakes in rural and remote Australia, a synthesis of the evidence from published studies that characterize dietary intake patterns in rural communities is needed.

Therefore, this systematic literature review aimed to (i) synthesize the evidence characterizing dietary intake among adults residing in rural and remote areas of Australia, (ii) identify key priority areas for intervention and policy, and (iii) make recommendations for future research. 

## 2. Materials and Methods 

### 2.1. Protocol and Registration 

**Figure 1:**
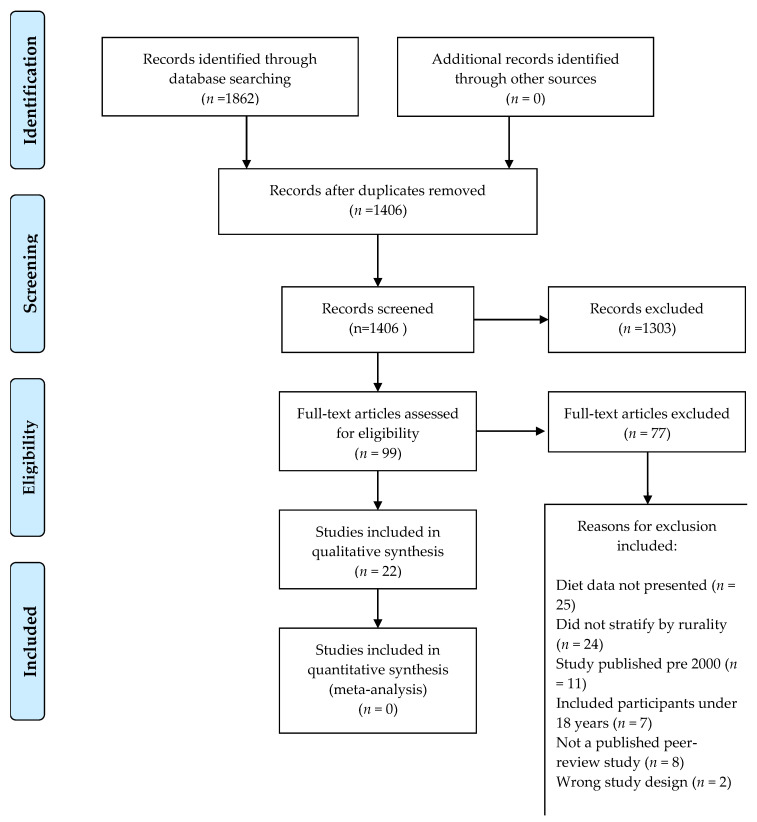
PRISMA flow diagram of included studies.

This systematic review was conducted and reported following the Preferred Reporting Items for Systematic Reviews and Meta-Analysis (PRISMA) statement guidelines [10] (See Supplementary Information Table S1 for checklist and Figure 1 for flow diagram). A protocol for the review was submitted to the International Prospective Register of Systematic Reviews (PROSPERO) (Registration number CRD42020173340).

### 2.2. Eligibility Criteria 

This review aimed to synthesize dietary intake data available from studies conducted in community settings outside of major cities of Australia (or MM2 and above, as classified by the Modified Monash Model (MMM)) [11], that included adults (defined as ≥18 years) defined by the Medical Subject Headings (MeSH) definitions. For ease, the term “rural” used throughout this paper refers to all areas classified as MM2 and above by the MMM [11].

Studies were included that met the following criteria:

- Study designs including
  ○ cross-sectional, longitudinal studies; and
  ○ randomised controlled trials, or before and after studies that included baseline dietary data;
- Dietary intake data including (but not limited to) serves of foods, food groups, and nutrient intake data collected using quantitative dietary assessment methods (e.g., FFQ, 24 h recall);
- All study settings were included (e.g., health care, community, home, or school-based settings) in areas classified as regional or remote based on the Australian Statistical Geographical Standard Remoteness Areas (ASGS-RA) [12] or categorized as MM2 or above [11]. If both rural and urban populations were included, dietary data must be stratified according to rurality;
- published in English due to a lack of translational resources;
- published on or after 1 January 2000 due to variation in the different remoteness classification systems being used over time [11,13].

Studies were excluded if they met the following criteria:

- Study designs including case reports, reviews, editorials, letters to the editor, or qualitative research;
- Inclusion of people under 18 years, or if people under 18 years of age were included but the authors did not stratify outcomes according to age;
- Inclusion of populations living in metropolitan areas only (MM2 and above) or if metropolitan and urban populations were included and the authors did not stratify outcomes according to rurality;
- Dietary intake was measured using qualitative methods, apparent consumption data, food supply data, or similar; or
- Reported dietary intake following an intervention or changes only (i.e., no baseline data).

### 2.3. Literature Search and Study Selection 

Five major electronic databases (CINAHL (EbscoHost), Medline (Ovid), EMBASE (Elsevier), Academic search premier (EbscoHost), and Rural and Remote Health database (INFORMIT), were systematically searched from 1 January 2000 until 30 April 2020 and online searching was conducted until 30 June 2020. 

Search terms included combinations, truncations, and synonyms of the following:

- Diet*; Nutrition*; Nutrient*; Macronutrient*; Energy; Fib*; Micronutrient*; Vitamin*; trace element*; Mineral*; Intake*
- regional Australia; remote Australia; remote; regional; farming community; community; New South Wales; Northern Territory; South Australia; Tasmania; Western Australia; Queensland; Victoria; Australian Capital Territory.

Additional articles were obtained through a manual search of reference lists, conference proceedings, and abstracts and by contacting experts in the field. 

An overview of the study selection is provided in Figure 1. One author (L.A.) carried out all electronic database searches, merged all search results into the reference management software (Covidence systematic review software; Veritas Health Innovation, Melbourne, Australia), and duplicate records were removed. Study selection followed the process described in the Cochrane Handbook of Systematic Reviews [14]. Two authors (L.A. and K.K.) independently screened all titles, abstracts, and full text articles to remove irrelevant studies according to the eligibility criteria. Any disagreements were discussed and resolved by consensus between two authors and there was no need for further consultation with a third author due to a high level of agreement between the two authors.

### 2.4. Data Extraction, Synthesis, and Quality Assessment

For studies meeting the inclusion criteria, information was extracted using a pre-designed electronic data extraction table that included details such as author, year of data collection, population and remoteness area (MMM) [11], number of participants, dietary data collection methods, dietary data characteristics, findings related to the dietary data, and strengths and limitations of the study. Additionally, it was determined whether the dietary outcomes were compared to or interpreted against recommendations made in the Australian Guide to Healthy Eating (AGHE) [15] and Australian Nutrient Reference Values (NRVs) [16]. Two authors extracted 50% of the data and conducted an independent cross-check of a random sample of 30% of the included studies for accuracy. When two or more articles reported results from the same study, all articles were considered together for complete data extraction. The researchers used the Australian Governments “Health Workforce Locator” (HWL) location classification database [17] to check the remoteness of each intervention based on information provided in the papers. If the location description was unclear, the lead author of the study was contacted to clarify the information in order to ensure correct remoteness classifications for both the ASGS and the MMM. If no response was received, an estimate of location remoteness was mapped using the HWL based on the information provided. 

The Newcastle Ottawa tool for cross-sectional data was used to assess the quality of each study [18]. Using this tool, each study was evaluated based on the appropriateness of the study design and the quality of how the study was conducted. The checklist allows an objective rating (positive, neutral, or negative) to be given to each study. The CREATE tool was used to assess the quality of studies including Indigenous participants and was assessed by TW (who identifies as an Aboriginal Australian) [19]. The CREATE tool is designed to operate alongside other quality appraisal tools. It does not score numerically and defines studies based on 14 questions that consider the cultural context and safety of conducting Indigenous research based on Indigenous ways of knowing.

The key characteristics of the included studies were summarized in text form and tabulated using the information collected from the data extraction form.

## 3. Results

As summarized in the PRISMA diagram in Figure 1, the searches retrieved 1862 abstracts in total and after the removal of duplicates, a total of 1406 articles were screened for inclusion based on their title and abstract. Of these, the full texts of 99 articles were reviewed to find 22 articles that met the inclusion criteria. Reasons for exclusion (Figure 1) at the full-text stage included that the studies did not report on dietary intake data as part of the manuscript results, included participants under the age of 18 years, and did not stratify results by rurality (if metropolitan participants were included). Full details of the study designs, method of diet data collection, results, and conclusions of the studies are summarized in Table 1. Of the 22 studies, almost half included dietary data that were 10 or more years old (collected in 2010 or earlier) [20,21,22,23,24,25,26,27,28]. Three of the studies were intervention studies and baseline dietary data were extracted only [21,29,30]. An overview of the distribution of study characteristics is provided in Table 2, including dietary outcomes reported and dietary assessment instrument used and whether the authors compared dietary intakes with national public health recommendations. 

### 3.1. Dietary Outcomes 

Dietary intake data were collected across all non-metropolitan classifications of the MMM, including areas classed as MM2-MM7, and most commonly in MM3 and MM4 (Table 2). Dietary data from a range of sample sizes were reported, ranging from 30–6020 people [28,41], most commonly with a sample size between 100–500 adults (Table 2). The studies presented the dietary data in a variety of ways. Most commonly, dietary data was reported as intake of food groups (e.g., grams or serves of fruit per day), followed by energy intake (total kJ/day) and information on macronutrient intake (in grams or kJ/day). Additionally, dietary data was presented using various diet quality scores, which compared dietary intake to adequacy of nutrient and/or food group recommendations. Non-quantifiable dietary data (i.e., without portion sizes) and micronutrient intake data were less commonly reported (Table 2).

#### 3.1.1. Food Groups

Nine studies reported dietary intakes in consumption of foods and food groups [26,27,29,33,37,39,40,42,43]. Fruit and vegetables were the most commonly reported food groups. However, a synthesis of the adequacy of dietary intakes of fruits and vegetables was not possible due to differences in the way studies reported dietary intake for these food groups (see Table 3 for differences in the reporting style for fruit and vegetables). Regardless, intakes of fruits and vegetables in rural populations were largely inadequate. For example, Nour et al. reported on the differences in intake between urban, regional, and remote young adults, showing that rural dwelling youth consumed less fruit and fruit juice and more starchy vegetables than their urban counterparts [43]. There was no significant difference in reported fruit and vegetable intake between people living in regional centers, large rural towns, and small rural towns in a cross sectional study of adults by Simmons et al. [20]. In this study, approximately half of respondents did not meet fruit intake recommendations and fewer (30%) respondents met vegetable recommendations. This study also reported that there was no relationship between eating takeaways monthly and risk of obesity [20]. Five studies presented non-quantifiable dietary intake data, most often reporting the frequency of consumption for particular foods (e.g., takeaway foods) (Table 2) [20,24,31,38,41].

#### 3.1.2. Nutrients

Eight studies reported on energy intakes and/or macronutrients intakes in their rural sample [21,22,29,30,34,35,36,40]. For example, Harrison et al. collected cross-sectional data in 2012–2013 from women (*n* = 649) residing in areas classed as MM3-MM6. The study described the mean nutrient intake of the sample, which was: mean energy intake of 7191 kJ/day, total fat intake of 74.3 g/day, saturated fat intake of 30.8 g/day, protein 88.0 g/day, carbohydrate 176.5 g/day, and 20.4 g of fiber per day [34]. Nutrient intakes in this sample were not compared with the AGHE or NRVs and instead, the associations between nutrient intakes and weight status were explored with the authors reporting that a higher BMI was associated with increased nutrient intakes [34]. Additionally, energy and macronutrient data was presented by Peach et al. for a sample of rural men (*n* = 131), showing that median energy intake was 11288 kJ/day, however dietary intakes were not compared with NRVs. Three studies reported micronutrient intake data (Table 2). Some studies were more targeted, reporting only micronutrient intakes in the absence of other dietary data. For example, Peach et al. 2000 used a self-administered quantitative food and beverage frequency questionnaire to determine calcium intake in 131 rural adults [23]. The authors presented dietary data as the proportion of respondents with low calcium intake against an identified cut off value, but did not report mean calcium intakes nor compare intake data clearly against the national NRVs [23]. 

#### 3.1.3. Dietary Patterns

Seven studies reported diet quality scores or applied dietary patterns analysis on dietary data collected in their sample of rural adults [24,26,28,30,32,35,36]. Thorpe et al., used an 111-item version of the Cancer Council FFQ in a cross-sectional study of 1667 adults (men and women) and benchmarked dietary intake using a score of compliance (The Dietary Guideline Index) with the AGHE [32]. The study found that in this large rural sample, participants demonstrated poor diet quality when compared with the AGHE recommendations [32] and that rural men (but not women) had significantly poorer diet quality when compared with urban respondents. Mishra et al. undertook a cross-sectional study with 6020 females aged 50–55 years using a 100-item version of the Cancer Council FFQ, analyzed the dietary data using factor analysis, and presented the results as daily frequency of consumption of 15 food groups [41]. The study compared diets between urban and rural women and found that the most frequently consumed foods for rural women were processed foods [25]. O’Kane applied a non-validated Food Habit Score to 10 food consumption questions in their cross-sectional study of Australian rural men as an indicator of diet-quality. Respondents to their survey with lower Food Habit Scores were significantly more likely to report needing a health scare before changing their lifestyle [24]. 

#### 3.1.4. Multiple Dietary Outcomes

Some studies collected high-quality dietary data and were able to report on multiple dietary factors. For example, Martin et al. (2018) presented baseline dietary data collected using the Cancer Council of Victoria FFQ from an RCT involving 230 females (aged 18–50 years) [30]. The study presented energy, macronutrient, and micronutrient intake and a score for diet quality using the “a priori” Dietary Guideline Index (DGI). Mean energy intake in the group was 8051.7 (SD 1827.6) kJ per day and diet quality scores were reported to be suboptimal at baseline [30]. Another study by Martin et al. (2017) presented cross-sectional data comparing the diets of rural women versus urban dwelling women [35]. Data presented showed that rural women consumed a mean of 7965.4 kJ of energy per day, 93.7 g of protein/day, 189.1 of carbohydrates per day, and 79.3 g of fat per day (41.1% being from saturated fat). The study did not benchmark the nutrient intakes against the NRVs, but hypothesized that women in rural areas had a higher meat intake than those in urban areas [35]. Lombard et al. collected dietary data as part of a randomised controlled trial at baseline, reporting energy intake (kJ/day) and daily intake (grams/day) of pre-defined food groups (fruit, vegetables, takeaway food, snack food, alcohol, and breakfast cereal). Although the study did not compare the reported dietary data with the AGHE, rural women in the sample were not consuming adequate fruit and vegetables according to AGHE recommendations [29].

### 3.2. Comparison with Public Health Nutrition Guidelines

Studies predominantly presented dietary data with no comparison against public health nutrition guidelines [20,22,24,28,30,31,34,35,37,40]. However, all of the studies that compared dietary intakes against public health nutrition guidelines showed that dietary intake was suboptimal in rural areas, except for one study, which found a high proportion of participants to be meeting the guidelines based on self-reported daily consumption of fruit and vegetables [33]. 

Nine studies compared food group intake to the Australian Guide to Healthy Eating (AGHE) [26,27,32,33,36,38,39,42,43] and only one study comprehensively interpreted dietary intakes against both the AGHE and NRVs [26]. For example, Brimblecombe et al. conducted a study with 148 Indigenous adults, using a culturally appropriate pictorial dietary questionnaire to understand patterns in fruit, vegetable, water, and soft drink intakes in a remote community [39]. Results were compared with recommendations in the AGHE and found that participants had low intakes of fruit and vegetables at baseline. Participants consumed an average of 75 g/day of fruit per day and 87 g/day of vegetables. Three other studies [26,29,43] also reported fruit and vegetable intakes in g/day, with each of these studies reporting that mean consumption fell below the recommendation of 300 g/day for fruits and 375 g/day for vegetables (Table 3). 

Other studies did not explicitly report dietary intakes in g/day or serves/day but reported the percentage of the study sample who met public health guidelines (Table 3). For example, Noble et al. reported that 84% of their sample (*n* = 377 participants attending an Aboriginal Community Controlled Health Service) reported consuming inadequate fruit or vegetables compared with AGHE recommendations [42]. Similarly, Burgis-Kasthala et al. asked 326 adult participants how many cups of fruit and vegetables they consumed on a daily basis and found that a high proportion of participants were meeting fruit and vegetable recommendations [33]. Overall, 47.2% of participants met the recommended daily fruit guidelines (38.8% of males and 51.9% of females) and 39.5% met the daily vegetable guidelines (33.6% of males and 42.8% of females) [33]. Xu et al. undertook a cross-sectional survey using a non-validated FFQ with 10 response options for a sample of Indigenous adults with type 2 diabetes. The study found that when compared to national recommendations for diet, both vegetable and fruit intake was very low, with no participants reporting adequate daily vegetable intake and only 10% reported adequate fruit intake [38]. The authors noted that if the data was representative of diet quality in Indigenous Australians with diabetes, this is poorer than that of the Indigenous population nationally [38].

Four studies compared dietary intakes against NHMRC Nutrient Reference Values (NRVs) [21,23,26]. Lee et al. used the 120-item Australian Eating Survey FFQ (AES FFQ) to assess dietary intake in a small sample (*n* = 58) of Indigenous pregnant women and applied the Australian Recommended Food Score (ARFS) to compare dietary intakes against the AGHE. The study found that none of the women met all recommendations and only a small percentage of the women (a third of women or less) were meeting AGHE recommendations across each food group [26]. A study by Rheinhardt et al. also assessed the diets of a small sample (*n* = 38) of pregnant women participating in a pilot intervention using a 74-item Victorian Cancer Council FFQ. The study provided key macronutrient and micronutrient information for the sample and showed that the rural women had suboptimal fiber recommendations and a high intake of saturated fat [21]. The pregnant women were consuming a mean of 8910 kJ/day, 90 g/day of fat, 38 g/day of saturated fats, and 223 g/day of carbohydrates in the intervention group. Owen et al., 2020 conducted a cross-sectional survey of rural adults aged 55–89 years (*n* = 458) using the AES FFQ. The authors applied the ARFS to provide a diet quality score for the sample [36]. The study reported that 50% of men and women did not meet recommended intakes of fiber and 60% of men and 42% of women exceeded recommended dietary sodium intakes.

### 3.3. Dietary Tools

A variety of dietary assessment techniques and tools were used including non-validated questionnaires or short surveys [20,22,23,24,27,28,31,33,37,38,39,42], followed by validated food frequency questionnaires [21,26,29,30,32,34,35,36,41] and 24 h food recalls [40,43]. 

Most studies included in this review used non-validated dietary assessment tools or short surveys with limited generalizability. An example of this is an intervention study conducted by Aoun & Rosenberg who reported baseline dietary intake data for 30 participants, which were collected using a short fat dietary score questionnaire [28], however was unclear how this score represents actual fat intake. Additionally, Lim et al. conducted a cross-sectional survey of 1154 adults and asked participants “on a typical day, how many servings of fruit/vegetable do you eat?” [37]. This is not a validated technique and the study did not specifically benchmark the outcomes against fruit and vegetable recommendations in the AGHE.

Nine studies used validated food frequency questionnaires [21,26,29,30,32,34,35,36,41], with the number of food items ranging from 10 to 120 items (Table 1). The Cancer Council Australia Food Frequency Questionnaire was the most commonly reported tool, with each study reporting consumption of various food groups (both core and non-core foods), nutrient intake data, and diet quality scores. In other studies, the food frequency questionnaire tools were inadequately described [22,23]. 

Two studies utilized a 24 h diet recall method, which is a comprehensive and well-validated dietary assessment technique that can allow for high-quality food group and nutrient information to be reported. For example, a cohort study that included 2583 Indigenous adults used brief interviews to ask participants to recall their dietary intake from the past 24 h. On average, participants reported consuming 1.0 serve of fruit in the past 24 h and 1.2 serves of vegetables [27]. Conversely, McMahon undertook a cross sectional 24 h recall survey with 1363 Indigenous participants from very remote communities and reported the contributions of food groups to overall energy intake [40]. 

### 3.4. Quality Assessment 

The methodological quality of the included studies varied (see Supplementary Tables S2–S4). Four studies received 5/10 stars [26,27,39,42], six studies [21,23,24,33,36,40] received 6/10 stars, and the remaining studies scored 7/10 stars or above (see Tables S2 and S3). The Aboriginal and Torres Strait Islander quality appraisal tool (CREATE) assessed six Indigenous-based studies [26,27,38,39,40,42] (Table S4). The majority of studies included either “unclear” or “no” answers to the CREATE 14 criteria, indicating room for culturally safe improvement in these papers, with a need to clearly report on study aspects such as “did the research demonstrate capacity strengthening for Aboriginal and Torres Strait Islander individuals”, “did the research have Aboriginal and Torres Strait Islander research leadership”, and “ensuring that the research is guided by an Indigenous research paradigm”.

## 4. Discussion

This systematic literature review assessed the evidence characterizing dietary intakes in rural Australian adults. The review highlights a paucity of information, with only 22 studies that have collected relevant dietary intake data in non-metropolitan populations in Australia in the past two decades and almost half of the studies including data that are 10 or more years old. More than eight million people reside in rural Australia, with substantial evidence of diet-related rural health inequalities that affect these individuals [1,3,4,8], yet this review demonstrates that there is very little understanding of dietary intake patterns in these areas.

Dietary intake data collected from rural populations was captured and presented in multiple ways which limited the possibility of pooling and synthesizing the dietary data in a more comprehensive way. Most commonly, dietary data was presented as consumption of food groups, namely intake of fruits and vegetables. However, there were inconsistencies in the way in which dietary data were collected and presented (Table 3), meaning it is not currently possible to consolidate or make comparisons between the studies, along with heterogeneity issues of the sampled populations. Only one study [26] provided a comprehensive overview of dietary intakes in their study sample, including both food group and nutrient analysis in addition to interpreting the dietary outcomes clearly against public health recommendations. Therefore, this publication could be useful to inform ideal reporting practices for future research. Less than half of the studies [21,23,26,32,33,36,39,42,43] benchmarked the dietary data collected with existing public health nutrition guidelines, such as the AGHE, adding to the challenge of making assessments of dietary intake between studies and among rural areas. These issues mean that with the data available to date, clear priority areas for potential initiatives to improve dietary intakes in rural Australia are unable to be generated. Consistency in dietary data collection is needed to identify specific needs amongst different populations residing across all levels of remoteness according to the MMM, along with an understanding of the drivers of dietary intakes in these areas. Such data would be used by researchers, local health services, health promotion officers, dietitians, local governments, and policy makers to understand nutrition priorities for their individual communities in reducing the burden of diet-related diseases. 

Overall, the studies showed that dietary intakes were suboptimal across all rural populations over the past 20 years, indicating that there is large potential for improvement in rural populations across Australia. While dietary data has been collected in relatively few rural Australian communities, it is likely that common challenges in rural food environments, such as poor food availability due to diminishing population sizes and lower food access as a result of needing to travel long distances to obtain food [44], mean that dietary intakes are likely to be suboptimal in most rural areas. Indeed, our investigation of fruit and vegetable intakes in the included studies (Table 3) highlights an average consumption between 87 and 199 g/day of fruit and 87 and 253 g/day of vegetables, which is substantially lower than public health recommendations (Fruit: 300 g/day and Vegetables: 375 g/day [15]). In line with previous research that shows that interventions targeting fruit and vegetable intakes should be the highest priority when seeking to reduce diet related chronic disease burden in rural Australian populations [4,8], only one study [33] showed a high proportion of participants were meeting fruit and vegetable guidelines. Almost half of the participants in this study met the recommended daily fruit guidelines and 39.5% met the daily vegetable guidelines. In contrast, data from the most recent National Nutrition Survey found that nationally, only 8% of adults reported meeting vegetable recommendations [45]. The authors explained that the discrepancy may be related to measurement error in the fruit and vegetable consumption data by using a non-validated, self-reported tool in addition to the fact that this study was conducted in a small sample in a single regional community that is not representative of the wider rural community. However, perhaps further research is warranted in this community to understand the drivers of higher fruit and vegetable intakes in a rural context. 

Half of the studies included in this review (50%) used non-validated questionnaires or short survey tools when collecting dietary intake data [20,22,23,24,27,28,31,33,37,38,39,42], indicating a major issue when interpreting and comparing the results. It is plausible that the use of non-validated tools and short surveys used in these studies are a result of low resourcing, along with considerations around ease for participants. Additionally, it may reflect that the chosen method was developed to meet the need of the individual study and provided relevant outcome measures for that specific research. It is well recognized that selecting a dietary assessment method that is valid and acceptable to both respondents and researchers can be challenging, especially for non-specialists [46]. While it is strongly recommended that dietary data is collected in collaboration with nutrition experts, toolkits for different research contexts are available for non-nutrition experts [47] to ensure high-quality dietary data is collected and presented. When validated tools were applied, FFQs were the most common dietary assessment technique [21,26,29,30,32,34,35,36,41] and were generally comprehensive tools with over 100 items. This reflects the broader literature and the common use of FFQ in research seeking to measure dietary intakes [48], despite the known measurement errors, mainly under-reporting of dietary intakes. FFQ has been shown to have higher measurement bias than the more accurate 24 h recall method, which was used by only two studies in this review [40,43]. The 24 h recall method is frequently used in study sub-samples to calibrate findings from the easier to administer FFQs [49]. We did not identify any studies in rural populations that validated the FFQ results in the study, alongside collecting intake data. Future dietary intake research in rural Australian populations must consider the accuracy of different dietary intake measures in the design of studies, with the inclusion of some form of sub-population validation assessment, as the majority of data synthesized in this review may be subject to high levels of error and could be an overestimation of the quality of dietary intake in rural areas. 

### 4.1. Recommendations for Future Research

In light of the findings of this review, it is important that researchers consider the implications around the scarcity of quality data collected on rural populations, the high levels of diet-related disease risks in these areas, and the potential future uses of dietary data that may lead to progress in addressing rural health disparities beyond the aims of individual studies. Where possible, collaborations should be established with nutrition professionals with expertise in dietary assessment methodologies to ensure valid, reliable tools are selected and that the outcome data is presented clearly, interpreted in the context of relevant research, and compared with national public health recommendations. A lack of high-quality dietary data collection and monitoring will contribute to inhibiting progress with the prevention of chronic disease in rural areas for future generations. Additionally, multidisciplinary rural health researchers should prioritize adding dietary outcomes to existing programs of health research, which could further our understanding of the environmental and/or health system factors relevant to diet-related disparities among rural populations. Future Australian research in rural communities should be conducted with representative populations; include standardized measures of rurality; use validated dietary assessment techniques; present comprehensive dietary outcome data; and clearly compare dietary intakes with relevant public health recommendations. 

### 4.2. Strengths and Limitations 

To our knowledge, this review is the first comprehensive synthesis of the literature using a systematic review methodology to synthesize the evidence on dietary intake data collected across rural Australia. A strength of this study is that we used broad search terms in multiple databases, across literature from the years 2000 to 2020. There are a number of limitations of this study, including that there was only a small number of highly heterogeneous studies that met the inclusion criteria, precluding a meta-analysis. As with all systematic reviews, the evidence synthesis here could be limited by publication bias, where studies with neutral or negative results may not be published, thus skewing results. Another limitation is that studies that included both rural and metropolitan populations but did not stratify results by remoteness were excluded, despite potentially showing efficacy and essential evidence for interventions in rural populations.

## 5. Conclusions

Despite the high level of preventable diet-related disease burden outside of major cities in Australia, there is a lack of high quality data available on the dietary intakes of rural dwelling adults to inform priorities for initiatives to improve dietary intake in these areas. Further and more frequent cross-sectional or longitudinal dietary data collection using robust dietary assessment tools is needed across all remoteness areas of Australia in order to adequately inform nutrition priorities and policy. Researchers need to consider the implications and potential future use of dietary data beyond individual studies to assist with progressing health and reducing diet-related chronic disease in rural areas.

## Figures and Tables

**Table 1:** Summary of included studies.

Author and Year	Setting	Rurality (MM) *	Age Group	Diet Questionnaire Type	Form of Data Presented	Diet Data Benchmarked against Dietary Guidelines	Summary of Results and Authors Interpretation
Aoun, S. & Rosenberg, M., 2004	Rehabilitation/secondary prevention, intervention study	MM2	Adults over 18 years *n* = 30	Short fat dietary score questionnaire	Short fat dietary score as a mean and SD	✘	Dietary fat intake (as a score) was high in this rural sample. Authors commented that scores were high at baseline.
Brimblecombe et al., 2018	Indigenous remote communities cross-sectional design	MM7	Adults over 18 years (*n* = 148)	Culturally appropriate pictorial diet questionnaire	Fruit, vegetable, water, and soft drink intake	✓	Only a small number of participants met guidelines for fruit and vegetable consumption. Participants generally had low intake of fruit and vegetables at baseline.
Burgis-Kasthala et al., 2019	Community based cross-sectional design	MM3-MM5	Adults over 18 years (*n* = 326)	Standardised lifestyle questionnaire	Fruit and vegetable intake (cups)	✓	A relatively high proportion of participants reported meeting AGHE recommendations. The proportion of rural people meeting recommendations in this sample was high and sample size was small.
D’Onise et al., 2012	Indigenous remote communities cross-sectional design	MM7	Adults over 18 years (*n* = 2583)	Questioned intakes from the past 24 h	Mean fruit and vegetable serves	✘	A large majority did not meet the fruit and vegetable recommendations. Recommended improved nutrition should continue to be a focus to reduce the life expectancy gap between Aboriginal and Torres Strait Islander people.
Harrison et al., 2017	Cross-sectional study	MM3-MM6	Women 18–50 years (*n* = 649)	Cancer Council FFQ	Macronutrients stratified by BMI	✘	Detailed macronutrient data presented but not interpreted. Recommended future initiatives should aim to improve health-related behaviors, with focus on optimizing social support and community engagement for obesity prevention in rural women.
Lee et al., 2018	Cross-sectional study	MM3	Pregnant Indigenous women (*n* = 58)	Australian Eating Survey FFQ	Mean serves of each food group, whether or not meeting recommendations and overall dietary score	✓	None of the participants met all of the AGHE recommendations. Almost all (93%) exceeded recommendations for discretionary foods. Further, 29.3% met vegetable recommendations and 27.6% met fruit recommendations. A low proportion met the NRV for iron. Only a small proportion of the women met recommendations for the AGHE.
Lim et al., 2017	Cross-sectional study	MM5	Adults over 18 years (*n* = 1154)	Fruit and vegetable intake- “on a typical day how many serves of fruit and vegetables do you eat?”	Intake of fruit and vegetables serves/day	✘	11% had 0 serves of fruit; 34% reported 2 serves of fruit/day. Vegetable intake 21% report 2 serves/day, 55% reported 3 or more. Higher fruit and vegetable intake were associated with older age, being female, and having private health insurance.
Lombard et al., 2016	RCT	MM2-MM6	Females 18–50 years (*n* = 649)	Cancer Council Australia Food Frequency Questionnaire (FFQ)	Energy intake (kJ/day), Fruit g/day, Vegetables g/day, Snack food g/day, Takeaway food, g/day; Bread g/day, Breakfast cereal g/day, Alcohol g/day	✘	Study showed change as a result of the intervention and did not benchmark with NRVs/RDIs but showed grams of fruit and vegetables that were below AGHE guidelines. Women in this sample were not meeting AGHE guidelines for fruit and vegetables.
Martin, et al., 2018	RCT	MM3-MM6	Females 18–50 years (*n* = 230)	Cancer Council Australia Food Frequency Questionnaire (FFQ)	Total diet quality and variety score	✘	Diet quality was suboptimal at baseline. Diet quality was improved by the low intensity intervention in rural women.
Martin et al., 2017	Cross-sectional	MM3-MM6	Females 18–50 years (*n* = 394)	Cancer Council Australia Food Frequency Questionnaire (FFQ)	Macronutrient and micronutrient intake	✘	Higher macronutrient consumption pattern in this sample of rural women was potentially related to a higher lean meat intake.
McMahon et al., 2017	Cross-sectional	MM7	Indigenous adults (*n* = 1363)	24 h recall	Food groups (percentage energy from major and sub-major food groups), Cereals & cereal products, Sugar products & dishes, Meat/poultry/game products & dishes, Non-alcoholic beverages	✘	Vegetable and fruit intakes only made up a small % of energy.
Mishra et al., 2005	Cross-sectional study	Large rural sample-Rurality of residence was not further defined by modified Monash model.	women aged 50–55 years (*n* = 6020)	Cancer Council of Victoria food frequency questionnaires	Mean frequency of consumption per day of Bread, Breakfast cereals, Pasta/noodles, Sweets biscuits, Fast foods. Snack foods, Sugar products and dishes, Milk/flavored milk, Cheese, Ice cream, Yoghurt, Nuts/peanut butter of paste, Chocolate, Vegemite, Meat, Poultry, Fish (steamed, grilled, or baked)	✘	There were differences in the dietary intakes between rural and urban women. The most frequently consumed foods for rural women were processed foods.
Noble et al., 2015	Cross-sectional	Regional/rural NSW-Rurality of residence was not further defined by modified Monash model.	Adults ≥18 years (*n* = 377 attending Aboriginal Community Controlled Health Service (ACCHS))	Fruit and Vegetable Consumption Two items; “How many serves of fruit/vegetables do you usually eat each day?”	<two serves of fruit; and/or <five serves of vegetables daily	✓	The relatively small variation in fruit and vegetable intake and under-screening across the sample suggests that almost all people attending an ACCHS would benefit from improved diet and screening.
Nour et al., 2017	Cross-sectional	MM2-MM6	Adults 18–34 years inner regional (*n* = 408) and outer regional and remote (*n* = 335)	24 h recall data	Intakes and variety of fruit and vegetables	✓	Fruit and vegetable intake was suboptimal among Australian young adults. Young adults consumed a mean of 0·9 and 2·7 servings of fruits and vegetables daily. Less than a quarter of the population surveyed reported consuming 3–4 different vegetable categories on the day prior to the dietary recall.
O’Kane et al., 2008	Cross-sectional	MM3-MM5	Males 25–64 years, (*n* = 529)	Food Habit Score survey	Food Habit Score, serves of Fruit; Vegetables; Cereal or bread products; Milk; Visible fat on meat; Butter or margarine on bread; Bread; Cheese; Cooking oil; Milk	✘	Participants received a mean Food Habit Score of 12/20, close to that achieved by urban and rural men in the Western Australia (WA) study (Food Habit Score of 12.4/20). The men in higher skilled occupations had a better diet quality than those from lower skilled occupations.
Owen et al., 2020	Cross-sectional	MM3 & MM4	Adults aged 55–89 years (*n* = 458)	120 item semi-quantitative food frequency questionnaire	The Australian Recommended Food Score (ARFS), a diet quality index that captures the dietary quality of key food groups, was calculated from the AES FFQ	✓	50% of men and women did not meet recommended intakes of fiber, while 60% of men and 42% of women exceeded recommended dietary sodium intakes.
Peach et al., 2002	Cross-sectional	MM2	Adult ≥18 years (*n* = 131)	Self-administered, semi-quantitative food and beverage frequency questionnaire	Energy (kJ/day); Starch (g/day); Sugars(g/day); Fats (g/day); Cholesterol (mg/day); Alcohol (g/day); Dietary fiber (g/day); Vitamin C (mg/day); Iron (mg/day); Calcium (mg/day); Coffee (cups/month); Tea (cups/month)	✘	Authors did not make specific conclusions about diet. Descriptive data only.
Peach et al., 2000	Cross-sectional	MM2	Adults ≥18 years (*n* = 332)	Self-administered semi-quantitative food and beverage frequency questionnaire	Assessed calcium intake only	✓	Low dietary calcium intake was highly prevalent in both males and females in this regional setting.
Reinhardt et al., 2012	Intervention study	MM3-MM5	Pregnant women ≥18 years (*n* = 38)	The Cancer Council Victoria Food Frequency Questionnaire (FFQ) was utilized and has 74 items grouped into four food categories	Mean energy intake (mean of intervention and control group) Energy (kJ/day); fat (g/day); saturated fat (g/day); fiber (g/day); Carbohydrate (g/day)	✓	High % of saturated fat. Fiber intake below recommendations.
Simmons et al., 2005	Cross-sectional	MM2-MM5	Adults over 25 years (*n* = 1454)	Validated questions from the Victorian population health survey	Takeaways less than 1/month Use full-fat milk Use low-fat spread Cut fat off the meat Cut skin off chicken Dairy items 2+/day Fruit 2+/day Vegetables 4+/day	✘	This study found that the reported dietary intake was not related to obesity/BMI in this rural sample. There was a high % of rural people meeting vegetable intakes compared to more recent national health survey data.
Thorpe et al., 2016	Cross-sectional	MM2-MM6	Australian adults aged 55–65 years; (*n* = 1667)	111-item food frequency questionnaire and additional food-related behavior questions.	Score for compliance with the Australian Dietary Guidelines- DGI-2013	✓	Adults aged 55–65 years demonstrated poor diet quality according to the DGI-2013
Xu et al., 2019	Cross-sectional	MM6-MM7	Indigenous adults ≥18 years with Type 2 diabetes (*n* = 210)	FFQ with 10 response options	Vegetables; Fruit Fresh fish; Milk-based Drinks; Juice; Coffee or tea; Water; Homemade Meals; Takeaway; Snacks; Diet soft drinks/cordials; Regular soft drinks/cordials; Alcohol	✘	Self-reported vegetable and fruit intake was very low; no participant reported adequate daily vegetable intake and only 10% reported adequate fruit intake. If representative of diet quality in Indigenous Australians with diabetes, this is poorer than that of the Indigenous population nationally, in which a greater proportion reported adequate vegetable and fruit intake (5% and 43%, respectively) and poorer than that reported for remote communities in the 2015 Health and Welfare of Australia’s Aboriginal and Torres Strait Islander peoples report. One in six reported consuming takeaways and 30% reported snacking at least twice weekly. This is slightly better than in the DRUID study of urban Indigenous Australians, 29% of whom reported consuming takeaway foods and 37% consumed snacks at least twice weekly. There was low fish intake, with only 4.3% meeting the CARPA guideline of two to three times per week.

AGHE = Australian Guide to Healthy Eating, MM = Modified Monash Model, NRV = nutrient reference values; * The Modified Monash Model measures remoteness and population size on a scale from MM 1 to MM 7, where MM 1 is a major city and MM 7 is very remote. A ✘ denotes ‘not present’, and a ✓ ‘present’.

**Table 2:** Characteristics of included studies.

**Characteristic**	**Number of Studies**		**References**
	Visual representation	Number of studies (%)	
Modified Monash Model classification			
MM2	••••••	6 (27)	[22,23,28,29,31,32]
MM3	•••••••••	9 (41)	[20,21,26,29,32,33,34,35,36]
MM4	•••••••••	9 (41)	[20,21,29,30,32,33,34,35,36]
MM5	••••	4 (18)	[20,21,29,30,32,33,34,35,37]
MM6	••••	4 (18)	[30,32,34,38]
MM7	•••••	5 (23)	[27,38,39,40]
Unclear but reported as rural and/or remote	•••••	5 (23)	[24,31,41,42,43]
Sample size (rural/remote sample only)			
<100	•••	3 (14)	[21,26,28]
100–500	•••••••••••	11 (50)	[22,23,30,31,33,35,36,38,39,42,43]
501–1000	••••	4 (18)	[24,29,32,34]
1001+	••••	4 (18)	[20,27,37,40]
Dietary outcomes			
Food group serves/grams	•••••••••	9 (41)	[26,27,29,33,37,39,40,42,43]
Macronutrient/s or energy	••••••••	8 (36)	[21,22,29,30,34,35,36,40]
Diet Quality Indices/Diet Score	•••••••	7 (32)	[24,26,28,30,32,35,36]
Non-quantifiable data (e.g., frequency of consumption)	•••••	5 (23)	[20,24,31,38,41]
Micronutrient/s	•••	3 (14)	[22,23,26]
Dietary tool			
Non-validated questionnaire or short survey	•••••••••••	11 (50)	[20,22,23,24,27,28,31,33,37,38,39,42]
Validated Food Frequency Questionnaire	•••••••••	9 (41)	[21,26,29,30,32,34,35,36,41]
24 h recall	••	2 (9)	[40,43]
Comparison to national public health recommendations			
None	••••••••••	10 (45)	[20,22,24,28,30,31,34,35,37,40]
Australian Guide to Healthy Eating	•••••••••	9 (41)	[26,27,32,33,36,38,39,42,43]
Nutrient Reference Values	••••	4 (18)	[21,23,26,36]

National Health and Medical Research Council (NHMRC) Australian Guide to Healthy Eating [15] and Nutrient Reference Values [16].

**Table 3:** Comparison of outcomes for fruit and vegetable intake and the dietary assessment tool used among the included studies.

**Reference**	**Fruit and Vegetable Consumption Reporting Style**				**Dietary Assessment Tool**
	**Mean or Median g/Day**	**Mean or Median Serves/Day**	**Mean or Median % Contribution to Energy/Day**	**% of Adequate or Inadequate Intake against Guidelines**	
[39]	Fruit: 75 g/day Vegetables: 87 g/day				Non-validated questionnaire or short survey
[33]				Meeting fruit guidelines: 47% Meeting vegetable guidelines: 39%	Non-validated questionnaire or short survey
[27]		Fruit 1.0 serves Vegetables 1.2 serves/day			Non-validated questionnaire or short survey
[26]	Fruit: 199.4 g/day Vegetables: 253.5 g/day	Fruit: 1.4 serves Vegetables: 3.4 serves/day		Meeting fruit guidelines: 16% Meeting vegetable guidelines: 17%	Validated Food Frequency Questionnaire
[37]				Fruit guidelines: 0 serves = 11%, 1 serve = 39%, 2 serves = 34%, 3 serves = 15%, Vegetable guidelines: 0 serves = 3%, 1 serve = 21%, 2 serves = 34%, 3 serves = 55%	Non-validated questionnaire or short survey
[29]	Fruit: 189 g/day Vegetables: 171 g/day				Validated Food Frequency Questionnaire
[40]			Fruit: 2.1% of energy Vegetables: 4.8% of energy		24 h recall
[42]				Inadequate fruit or vegetable intake: 84%	Non-validated questionnaire or short survey
[43]	Fruit: 128 g/day Vegetables: 205 g/day	Fruit: 0.9 serves/day Vegetables: 2.7 serves/day			24 h recall
Australian Public Health Recommendation [15]	Fruit: 300 g/day Vegetables: 375 g/day	Fruit: 2 serves Vegetables: 5 serves	-	**-**	

## Supplementary Materials

The following are available online at https://www.mdpi.com/2072-6643/12/11/3515/s1, Table S1. Preferred Reporting Items for Systematic Reviews and Meta-Analyses (PRISMA) Checklist; Table S2. Non-Indigenous studies quality assessment; Table S3. Indigenous based studies quality assessment; Table S4. CREATE quality assessment tool.
