# Supplementary material for: Characterizing Dietary Intakes in Rural Australian Adults: A Systematic Literature Review
Source: Nutrients. 2020 Nov 15;12(11):3515. doi: 10.3390/nu12113515 (PMC7697691; doi:10.3390/nu12113515)
Supplement: Supplementary file 1 [file nutrients-12-03515-s001.pdf]

Supplementary Tables

Table S1 - Preferred Reporting Items for Systematic Reviews and Meta-Analyses (PRISMA) Checklist

Section/topic	#	Checklist item	Reported on page #
**TITLE**			
Title	1	Identify the report as a systematic review, meta-analysis, or both.	1
**ABSTRACT**			
Structured summary	2	Provide a structured summary including, as applicable: background; objectives; data sources; study eligibility criteria, participants, and interventions; study appraisal and synthesis methods; results; limitations; conclusions and implications of key findings; systematic review registration number.	1
**INTRODUCTION**			
Rationale	3	Describe the rationale for the review in the context of what is already known.	2
Objectives	4	Provide an explicit statement of questions being addressed with reference to participants, interventions, comparisons, outcomes, and study design (PICOS).	2
**METHODS**			
Protocol and registration	5	Indicate if a review protocol exists, if and where it can be accessed (e.g., Web address), and, if available, provide registration information including registration number.	2
Eligibility criteria	6	Specify study characteristics (e.g., PICOS, length of follow-up) and report characteristics (e.g., years considered, language, publication status) used as criteria for eligibility, giving rationale.	2, 3
Information sources	7	Describe all information sources (e.g., databases with dates of coverage, contact with study authors to identify additional studies) in the search and date last searched.	3
Search	8	Present full electronic search strategy for at least one database, including any limits used, such that it could be repeated.	3
Study selection	9	State the process for selecting studies (i.e., screening, eligibility, included in systematic review, and, if applicable, included in the meta-analysis).	3
Data collection process	10	Describe method of data extraction from reports (e.g., piloted forms, independently, in duplicate) and any processes for obtaining and confirming data from investigators.	3, 4

Data items	11	List and define all variables for which data were sought (e.g., PICOS, funding sources) and any assumptions and simplifications made.	Table 1
Risk of bias in individual studies	12	Describe methods used for assessing risk of bias of individual studies (including specification of whether this was done at the study or outcome level), and how this information is to be used in any data synthesis.	4
Summary measures	13	State the principal summary measures (e.g., risk ratio, difference in means).	N/a
Synthesis of results	14	Describe the methods of handling data and combining results of studies, if done, including measures of consistency (e.g., I^2) for each meta-analysis.	N/a
Section/topic	#	Checklist item	Reported on page #
Risk of bias across studies	15	Specify any assessment of risk of bias that may affect the cumulative evidence (e.g., publication bias, selective reporting within studies).	n/a
Additional analyses	16	Describe methods of additional analyses (e.g., sensitivity or subgroup analyses, meta-regression), if done, indicating which were pre-specified.	n/a
**RESULTS**			
Study selection	17	Give numbers of studies screened, assessed for eligibility, and included in the review, with reasons for exclusions at each stage, ideally with a flow diagram.	Figure 1
Study characteristics	18	For each study, present characteristics for which data were extracted (e.g., study size, PICOS, follow-up period) and provide the citations.	Table 1
Risk of bias within studies	19	Present data on risk of bias of each study and, if available, any outcome level assessment (see item 12).	Supplementary tables S2, S3, S4
Results of individual studies	20	For all outcomes considered (benefits or harms), present, for each study: (a) simple summary data for each intervention group (b) effect estimates and confidence intervals, ideally with a forest plot.	n/a
Synthesis of results	21	Present results of each meta-analysis done, including confidence intervals and measures of consistency.	n/a
Risk of bias across studies	22	Present results of any assessment of risk of bias across studies (see Item 15).	n/a
Additional analysis	23	Give results of additional analyses, if done (e.g., sensitivity or subgroup analyses, meta-regression [see Item 16]).	n/a

**DISCUSSION**			
Summary of evidence	24	Summarize the main findings including the strength of evidence for each main outcome; consider their relevance to key groups (e.g., healthcare providers, users, and policy makers).	5
Limitations	25	Discuss limitations at study and outcome level (e.g., risk of bias), and at review-level (e.g., incomplete retrieval of identified research, reporting bias).	5
Conclusions	26	Provide a general interpretation of the results in the context of other evidence, and implications for future research.	6
**FUNDING**			
Funding	27	Describe sources of funding for the systematic review and other support (e.g., supply of data); role of funders for the systematic review.	6

Table S2 - Non-Indigenous studies quality assessment

	**Aoun et al. 2004**	**Burgis-Kasthala et al. 2019**	**Mishra et al. 2005**	**Harrison et al. 2017**	**Lim et al. 2017**	**Lombard et al. 2016**	**Martin et al. 2018**	**Martin et al. 2017**	**Nour et al. 2017**	**O'Kane et al. 2008**	**Owen et al. 2020**	**Peach et al. 2002**	**Peach et al. 2000**	**Reinhardt et al. 2012**	**Simmons et al. 2005**	**Thorpe et al. 2016**
**Selection (Max 5 stars)**																
1) Representativeness of the sample:	B (Was regional but not remote considerate)	B	B* (Nationwide random selections)	A (Random town selections all with rural definitions)	B	A	A	B	B (Regional and urban)	A	B (Focused on older populations not entire ones in regional areas)	B (Men only, regional city only)	B	B	A (Truly representative based on study criteria)	B (Uses older people and rural included but not strictly rural)
2) Sample Size	B (Not many details given about the cross-sectional	B (Not enough despite the spread of participants	A	A	A (Large size for area it included)	A	A	A	A	A	A	B	A	B (Only 38 women, was hard due to	A	A

	sample)	from regional areas)												gestational diabetes focus)		
3) Non-respondents	A (This was very clearly outlined)	B	B (Could not follow up non-responders due to confidentiality reasons)	C	C	B (Discusses predictions in both groups and reflective of why responses might be different but doesn't mention on non-responder	B	B	C	B (Poor rate of return at 27%)	B (Another poor total response rate and no mention of non-respondents)	B (Good response rate but no mention of non-responders)	B (Reasonable response rates at 67% but no mention of non-responders)	B (No mention of non responders but reasonable response rate)	A (Accounts for all participants and reason for non responses also)	B (Response rate reported at only 38% and no take on non-responders)

						differ ence)										
4) Ascertain ment of the exposure (risk factor)	A	A	A^	A*	A	A	A	A	A	B	A	A (Serum ferritin measur es contrast ed with serum glucose /TAGs)	B (Used a FFQ but weren 't specifi c as to the one they used validit y only reliabi lity)	A	A (Use of objectiv e and subjecti ve measur es to validat e obesity includi ng BMI, WC and self-reporte d measur es)	A (Note s conve rgent validit y with stand ard dietar y guidel ines and use of the FFQ)
**Comparability (Max 2 stars)**																
1) The subjects in different outcome groups	A	A	B (Adjust ed for socio-demogr	A (Adjust ed for major individ	A & B*	A*	A*	B (Con trols man y	A	A	A	A	A	A	A & B (Measu res all other socio-	A

are comparable, based on the study design or analysis. Confounding factors are controlled.			aphic variables)	ual, social and environmental factors where it could)				things but rural to metro difference was a point of this paper)							demographic variables as well as obesity measures)	
**Outcome (Max 3 stars)**																
1) Assessment of the outcome	B	C	C	C (Did most of this via self-report)	C	A	A	A	C	C	C (used lots of objective lab testing but primary outcome was food and	A	C (Self-report on dietary calcium intakes assessed from FFQ)	C	C* (Assumes take-away food by self report to determine adiposity and this self-	C (Extensive 111 item FFQ but self report based still)

											based on an FFQ)				reporting for major outcome may not be truly accurate)	
2) Statistical test	A	A	A	A	A	A	A	A	A	A (Used p-values but was a little vague in description)	A	A	A	A	A	A
Notes:			* Said B due to it not being specific to rural, included metro. ^ Uses	* Uses mix of valid and mentioned tools that aren't so	* Used multivariate analyses to control for confounders but	* Used multiple measuring methods, BMI argua	* As per Lombard paper.								* Sparing self-report data for the aim of the study, based on this	

			cancer council FFQ	scored as A but could be a B?	may just be B however it answers itself in the aim so included A also.	bly most effective measuring tool WRT this study.									criteria it was nearly a 10/10.	
**Score (Stars):**	8/10	6/10	7/10	7/10	8/10	9/10	9/10	8/10	7/10	6/10	6/10	7/10	6/10	6/10	9/10	7/10

Details on scoring using the Newcastle-Ottawa Scale can be found at http://www.ohri.ca/programs/clinical_epidemiology/nosgen.pdf

Table S3. Indigenous based studies quality assessment

	Brimblecombe et al. 2018	D'Onise et al. 2012	Lee et al. 2018	McMahon et al. 2017	Noble et al. 2015	Xu et al. 2019
**Selection (Max 5 stars)**						
1) Representativeness of the sample:	**B**	**B**	**C** (Pregnant women only)	**B** (Uses very remote data only)	**B**	**B**
2) Sample Size	**B**	**B**	**B**	**A***	**B**	**B**
3) Non-respondents	**B***	**B***	**C**	**B** (Good response rate but no comparison mentioned for non-respondents or use of different surveys confounded things)	**C**	**A** (Did a non-responders analyses and were still able to make findings more generalisable)
4) Ascertainment of the exposure (risk factor)	**B**	**A**	**A**	**B** (Food and beverage purchasing as a surrogate doesn't give direct intake data i.e food waste etc.)	**B**	**A*** (Tools were validated or semi-validated)
**Comparability (Max 2 stars)**						
1) The subjects in different outcome	**B** (other confounders were	**N/A** (No controls and residual &	**A***	**B** (Comparison groups were	**A**	**B**

groups are comparable, based on the study design or analysis. Confounding factors are controlled.	considered)	unmeasured confounding)		different and controlling for it too difficult)		
**Outcome (Max 3 stars)**						
1) Assessment of the outcome	**C** (quantified but was self-reported data)	**B** (record-linkages from Well-Persons Health Check)	**C** (Does use valid and reliable FFQ but is self-report nonetheless)	**C**	**C**	**C** (Was all based on self-report)
2) Statistical test	**A**	**A**	**A** (No p-value but use of CI and IQR to measure food intake and daily serves relevant)	**A**	A	A
Notes:	* Scored as B because some description of changes were given even though there was no separation of respondents.	* High attrition & sample not representative at follow up (younger, AOD users etc.)	* No direct comparison group but compared to Aus Guide to Healthy Eating and the NRV's)	* Large sample of very remote Indigenous People (n = 1,363)	N/A	* Validated tools for assessment but some were not entirely culturally valid or gender valid to distinct from men's or women's business etc.
Score	5/10	5/10	5/10	6/10	5/10	7/10

Details on scoring using the Newcastle-Ottawa Scale can be found at http://www.ohri.ca/programs/clinical_epidemiology/nosgen.pdf

Table S4. CREATE quality assessment tool

	**Xu et al. 2019**	**McMahon et al. 2017**	**Brimblecombe et al. 2018**	**D'Onise el at. 2012**	**Lee et al. 2018**
1. Did the research respond to a need or priority determined by the community?	No	Partly	Partly	Partly (more indirectly through non-smoking and decreased alcohol and increased F&V intake not red cell folate directly)	Yes
2. Was community consultation and engagement appropriately inclusive?	Partly (consulted with Central Australian Aboriginal Congress & Menzies School of Health Research)	No	Partly	Partly (notes support from relevant Indigenous health bodies but doesn't specify)	Yes
3. Did the research have Aboriginal and Torres Strait Islander research leadership?	Unclear	No	No	No	Partly
4. Did the research have Aboriginal and Torres Strait Islander governance?	Partly (based on the acknowledgements section)	Unclear	Yes	No	Partly (Acknowledged an ongoing Indigenous steering committee)

5. Were local community protocols respected and followed?	Yes (use of interpreters in the local Alice Springs area)	Unclear	Yes	Unclear	Partly
6. Did the researchers negotiate agreements in regards to rights of access to Aboriginal and Torres Strait Islander peoples’ existing intellectual and cultural property?	No	Unclear	Partly	Unclear	Unclear
7. Did the researchers negotiate agreements to protect Aboriginal and Torres Strait Islander peoples' ownership of intellectual and cultural property created through the research?	Unclear	Unclear	Partly	Unclear	Unclear
8. Did Aboriginal and Torres Strait Islander people and communities have control over the collection and management of research materials?	No	No	Unclear	Unclear	Unclear
9. Was the research guided by an Indigenous research paradigm?	No	No	No	No	No
10. Does the research take a strength-based approach, acknowledging and moving beyond practices that have harmed Aboriginal and Torres Strait Islander peoples in the past?	Unclear	No	Partly	No	Partly (It is at least acknowledged in this paper)
11. Did the researchers plan to translate the findings into sustainable changes in	Partly	Partly	Yes	Partly	Partly

policy and/or practice?					
12. Did the research benefit the participants and Aboriginal and Torres Strait Islander communities?	Partly	Partly	Yes	Partly	Partly (Advocacy for more specific nutrient and food recommendations for pregnant Indigenous women)
13. Did the research demonstrate capacity strengthening for Aboriginal and Torres Strait Islander individuals?	No	No	Partly	No	Partly
14. Did everyone involved in the research have opportunities to learn from each other?	Unclear	Unclear	Unclear	Unclear	Unclear

Details on the scoring criteria for each study using the Aboriginal And Torres Strait Islander Quality Appraisal Tool can be found at https://doi.org/10.1186/s12874-020-00959-3
